# Effects of SIPA1L1 on trabecular meshwork extracellular matrix protein accumulation and cellular phagocytosis in POAG

**DOI:** 10.1172/jci.insight.174836

**Published:** 2024-11-22

**Authors:** Chenyu Xu, Jiahong Wei, Dan Song, Siyu Zhao, Mingmin Hou, Yuchen Fan, Li Guo, Hao Sun, Tao Guo

**Affiliations:** 1 Department of Ophthalmology, Ninth People’s Hospital, Shanghai Jiao Tong University School of Medicine, Shanghai, China.; 2 Shanghai Key Laboratory of Orbital Diseases and Ocular Oncology, Shanghai, China.; 3 Bengbu Medical University, Bengbu, China.; 4 Department of Ophthalmology, Songjiang Hospital, Shanghai Jiao Tong University School of Medicine, Shanghai, China.; 5 Department of Ophthalmology, Tongji Hospital, Tongji University School of Medicine, Shanghai, China.; 6 Department of Ophthalmology, First Affiliated Hospital of Bengbu Medical College, Bengbu, China.; 7 Lu’an Hospital Affiliated to Anhui Medical University, Lu’an, China.; 8 Lu’an People’s Hospital, Lu’an, China.

**Keywords:** Ophthalmology, Extracellular matrix

## Abstract

Accumulation of extracellular matrix (ECM) proteins in trabecular meshwork (TM), which leads to increased outflow resistance of aqueous humor and consequently high intraocular pressure, is a major cause of primary open-angle glaucoma (POAG). According to our preliminary research, the RapGAP protein superfamily member, signal-induced proliferation-associated 1-like 1 protein (SIPA1L1), which is involved in tissue fibrosis, may have an impact on POAG by influencing ECM metabolism of TM. This study aims to confirm these findings and identify effects and cellular mechanisms of SIPA1L1 on ECM changes and phagocytosis in human TM (HTM) cells. Our results showed that the expression of SIPA1L1 in HTM cells was significantly increased by TGF-β2 treatment in label-free quantitative proteomics. The aqueous humor and TM cell concentration of SIPA1L1 in POAG patients was higher than that of control. In HTM cells, TGF-β2 increased expression of SIPA1L1 along with accumulation of ECM, RhoA, and p-cofilin 1. The effects of TGF-β2 were reduced by si-SIPA1L1. TGF-β2 decreased HTM cell phagocytosis by polymerizing cytoskeletal actin filaments, while si-SIPA1L1 increased phagocytosis by disassembling actin filaments. Simultaneously, overexpressing SIPA1L1 alone exhibited comparable effects to that of TGF-β2. Our studies demonstrate that SIPA1L1 not only promotes the production of ECM, but also inhibits its removal by reducing phagocytosis. Targeting SIPA1L1 degradation may become a significant therapy for POAG.

## Abstract 2

SIPA1L1 not only promotes the production of ECM, but also inhibits its removal by reducing phagocytosis.

## Introduction

As a leading irreversible blindness disease worldwide, glaucoma threatens human vision health seriously (1). It is predicted that the number of glaucoma patients will grow to 111.8 million in 2040, and more than 74% of them have primary open-angle glaucoma (POAG) (2, 3). Intraocular pressure (IOP) stands as the sole modifiable risk factor, while other significant risk factors encompass advanced age, non-White ethnic background, and a positive familial predisposition to glaucoma (4–6). Glaucoma leads to optic nerve and retina damage and eventually irreversible blindness if the patient’s IOP is not effectively controlled (7). IOP elevation is a result of increased outflow resistance of the aqueous humor. Approximately 85% of aqueous humor leaves the eye through the trabecular meshwork (TM) and the rest exits via non-TM routes, including the uveoscleral outflow pathway (8–10). Blockade of the TM pathway by an excessive accumulation of extracellular matrix (ECM) proteins is believed to be a main cause of reduced aqueous humor outflow, which involves abnormal metabolism of ECM and probably a reduced phagocytotic function of the TM cells (11, 12).

At present, POAG is managed by IOP reduction, either via medications or surgical procedures. However, none of these treatments directly address the underlying mechanism of pathogenesis — the excessive ECM accumulation in the TM (13, 14). Therefore, it is an urgent unmet medical need to explore and understand the cellular and molecular factors related to ECM metabolism and TM functions.

Signal-induced proliferation-associated 1-like 1 (SIPA1L1), a member of signal-induced proliferation-associated (SIPA; also known as spine-associated RapGAPs, SPAR) protein family, is part of the Rap GTPase-activating proteins (RapGAPs) superfamily. The SIPA family consists of 4 members, SIPA1, SIPA1L1, SIPA1L2, and SIPA1L3, and all SIPA family members possess the following characteristic functional domains: an N-terminal RapGAP, a PSD-95/Dlg/ZO-1 (PDZ), and a C-terminal coiled-coil domain carrying a leucin zipper motif (15–19). SIPA1L1 has an additional domain of unknown function (DUF340), 2 additional actin-regulated domains (Act1 and Act2), as well as a guanylate kinase–binding domain (GKBD), which regulates the excitability of hippocampal neurons (15, 20). It has been reported that the RapGAP domain of SIPA1L1 promotes hydrolysis of Rap1-bound GTP to GDP, thereby reducing the activity of Rap1 (21). The PDZ domain of SIPA1L1 plays a key role in activating Rap1GAP. A recent report has provided evidence showing that SIPA1L1 binds to the COOH terminus of aquaporin 2 (AQP2) in the renal collecting duct cells and activates the RapGAP domain (22). The interaction of the PDZ domain and Ephrin receptor family member Epha4 also promotes phosphorylation of SIPA1L1 and inactivation of the small GTPases Rap1 and Rap2 (16, 20, 23).

The inactivated Rap1 has no capacity to inhibit the activity of Rho (a member of small G protein superfamily) (24). The RhoA protein, which belongs to Ras-related small GTP-binding proteins, plays an indispensable role in the expression of ECM and cell actin dynamics (25–27). Recent studies indicated that inhibition of RhoA can promote phagocytosis in TM cells (28, 29), since RhoA is capable of accelerating the phosphorylation of cofilin 1 (CFL1), which prevents polymeric filamentous actin (F-actin) from being broken down into monomeric globular actin (G-actin), leading to accumulation of F-actin (25–27, 30, 31). Therefore, SIPA1L1 is expected to affect ECM metabolism and phagocytosis.

In the liver, it has been reported that SIPA1L1 is an early indicator for liver fibrosis (32). In the eye, SIPA1L1 is extensively expressed in early *Xenopus laevis* development, especially in the optic cup and lens (15). Recent reports have revealed an upregulation in SIPA1L1 expression within TM cells in response to dexamethasone, a widely recognized IOP-elevating agent (33, 34). However, the regulatory roles of the SIPA1L1 protein are unknown in human TM (HTM) cells.

In this study, we found that the SIPA1L1 level, similarly to that of TGF-β2, was higher in aqueous humor samples of POAG patients. In cultured HTM cells, we found that TGF-β2 upregulated the expression of SIPA1L1, and that knockdown of SIPA1L1 reduced TGF-β2–induced ECM expression and phagocytosis. These results suggest that SIPA1L1 is an important factor in regulating the biological functions of HTM cells, which in turn has significant implications in the pathogenesis of POAG and its treatment.

## Results

### Label-free quantitative proteomics detected excessive expression of SIPA1L1 protein in HTM cells after TGF-β2 treatment.

**Figure 1:**
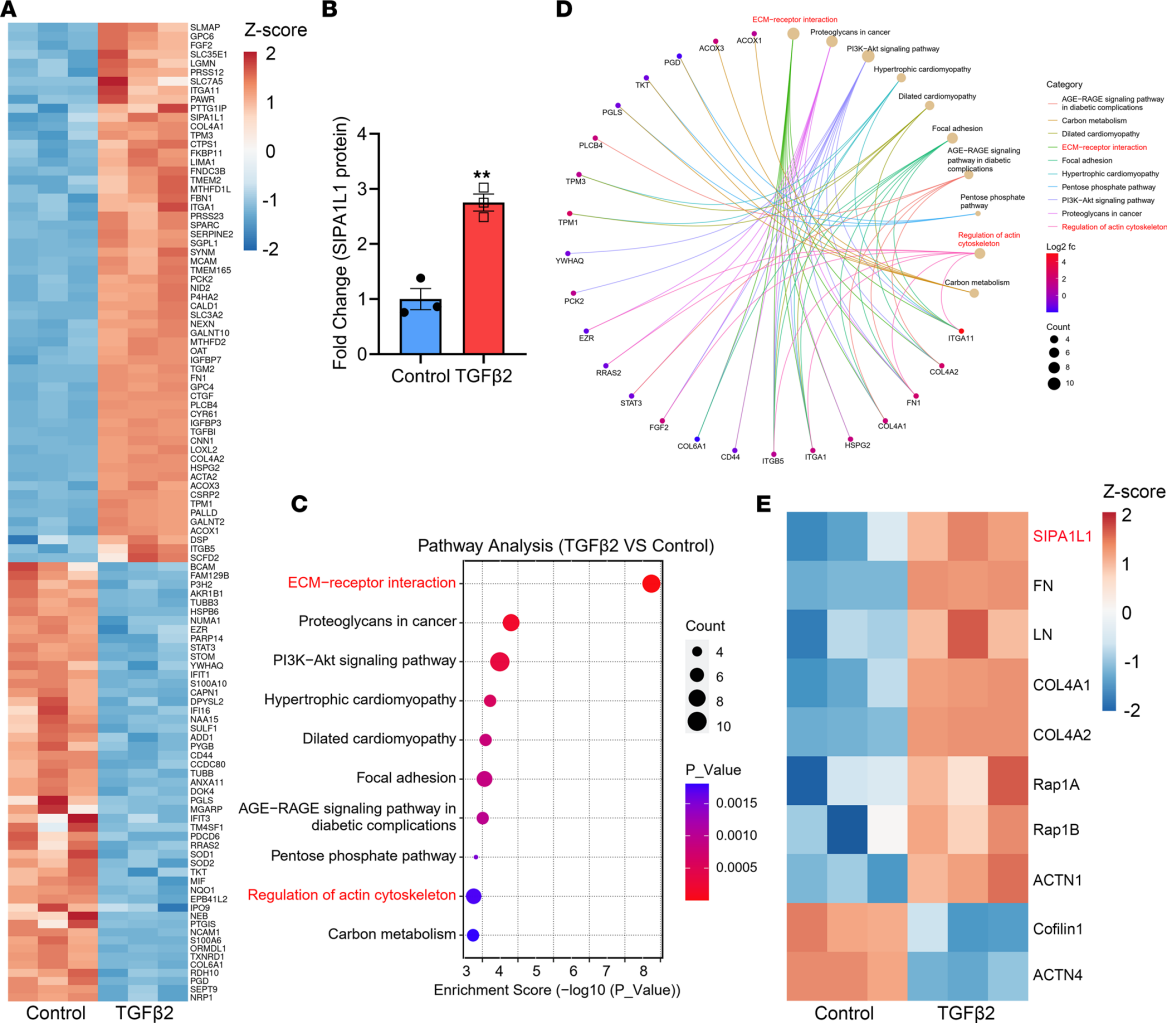
Label-free quantitative proteomics identifies SIPA1L1 protein is enriched in HTM cells after TGF-β2 treatment. HTM cells were treated with TGF-β2 (5 ng/mL) for 48 hours (TGFβ2 group) or without (control group), and the lysates were tested by label-free quantitative proteomics. The experiment used 3 individual HTM cell strains. (**A**) A total of 110 proteins were identified with greater than 2-fold differences and *P* < 0.05 between 2 groups according to protein clustering analysis. Red: Higher expression levels. Blue: Lower expression levels. Number: Intensity of each protein normalized by *z* score. (**B**) Levels of SIPA1L1 in control and TGF-β2–treated HTM cells. Data are expressed as mean ± SEM (*n* = 3). (**C**) The top 10 statistically significant enriched KEGG pathways in TGF-β2–treated cells, in which the ECM-receptor interaction and regulation of actin cytoskeleton are the most prominent (highlighted in red). Count: The number of proteins corresponding to the pathway. (**D**) Pathways associated with the upregulated proteins and downregulated proteins. Log2 fc, the fold change of each protein transformed by log_2_. (**E**) After analyses of protein clustering and KEGG, differentially expressed proteins that are closely relevant to ECM accumulation and actin cytoskeleton regulation in TGF-β2–treated HTM cells were determined. **P < 0.01 compared with controls by unpaired, 2-tailed Student’s *t* test.

We used label-free quantitative proteomics to explore proteins that were shown to affect levels of ECM and phagocytic function in HTM cells. HTM cells were serum-starved for 24 hours and then treated with TGF-β2 (5 ng/mL) for 48 hours. A total of 2734 proteins were identified, out of which 110 proteins were classified as differentially expressed, exhibiting an expression difference greater than 2-fold (up- or downregulation) and a *P* value of less than 0.05. Figure 1A shows that, among the 110 proteins, approximately 60% were upregulated and 40% were downregulated after TGF-β2 treatment. Analysis of protein clustering showed that SIPA1L1 was enriched in HTM cells with TGF-β2 induction compared with the control (fold change > 2.5, *P* = 0.0021; Figure 1B). Kyoto Encyclopedia of Genes and Genomes (KEGG) analysis indicated that TGF-β2 treatment affected multiple metabolic and signal transduction pathways in HTM cells, in which ECM-receptor interaction and regulation of actin cytoskeleton were identified (Figure 1, C and D). On the bases of the SIPA1L1’s function for RapGAP, combined with the analyses of protein clustering and KEGG, we discovered that Rap1A, Rap1B, and ACTN1 were significantly increased by TGF-β2 induction, while CFL1 and ACTN4 were markedly decreased (Figure 1E). These results indicate that SIPA1L1 expression in the HTM cells was increased by the treatment with TGF-β2, a cytokine known to be associated with POAG.

### Levels of TGF-β2 and SIPA1L1 were higher in TM cells and aqueous humor of patients with POAG.

**Figure 2:**
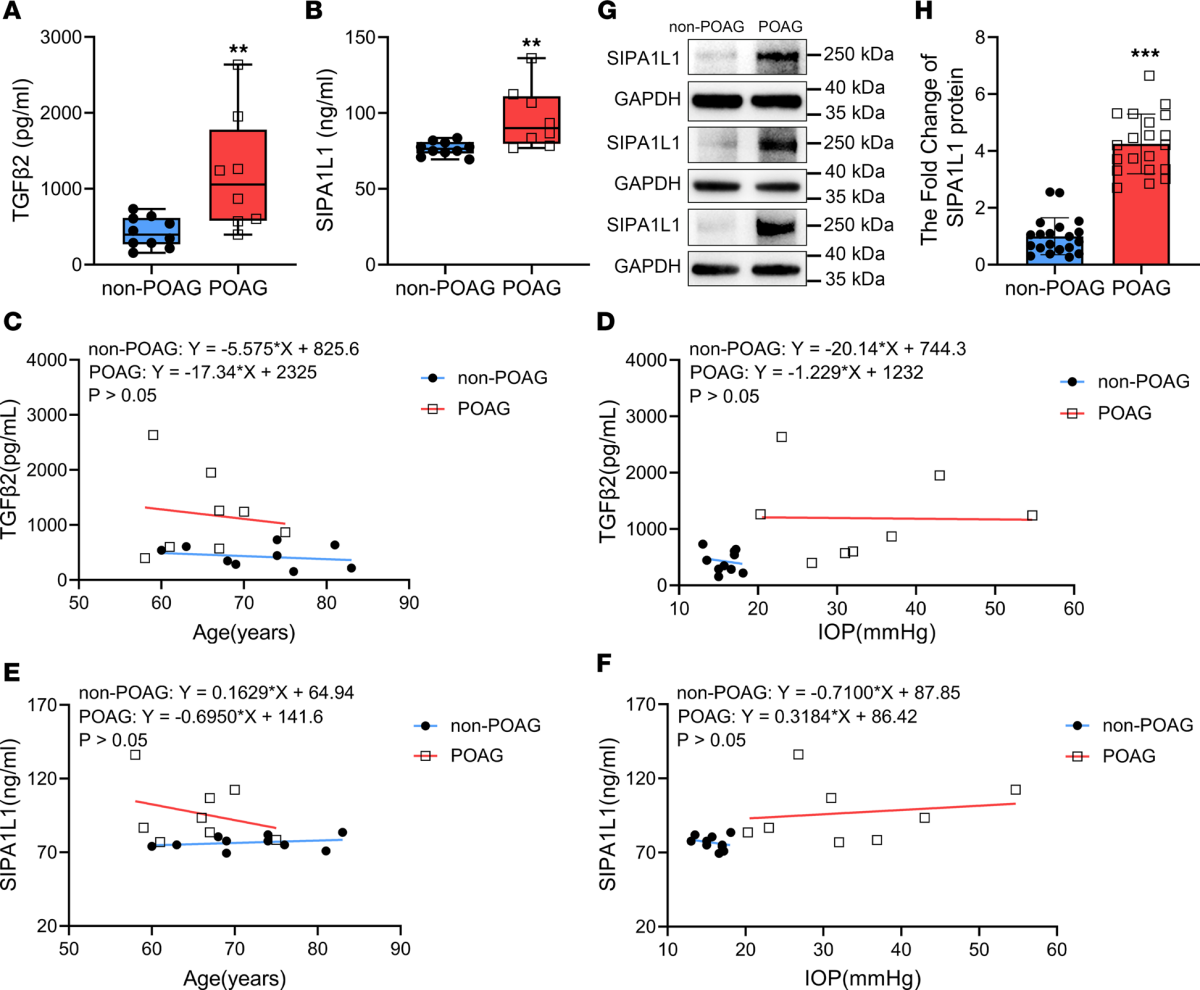
The concentration of SIPA1L1 is highly expressed in TM cells and aqueous humor from POAG patients. (**A** and **B**) Aqueous humor concentrations of TGF-β2 and SIPA1L1 in non-POAG (*n* = 10) and POAG patients (*n* = 8) were assayed by ELISA. (**C** and **D**) Aqueous humor levels of TGF-β2 had no significant correlations with age or IOP within the non-POAG and POAG patients. (**E** and **F**) Similarly, within the non-POAG and POAG patients, SIPA1L1 levels did not correlate with age or IOP. (**G** and **H**) TM cell concentration of SIPA1L1 in non-POAG (*n* = 3) and POAG patients (*n* = 3) was analyzed by Western blotting. Data are expressed as mean ± SEM (*n* = 3–10). **P < 0.01, ***P < 0.001 compared with non-POAG groups by unpaired, 2-tailed Student’s *t* test. Each symbol in graphs represents the data from each experiment.

To try to corroborate the observations in HTM cells, we used Western blotting and ELISA to assess the concentrations of SIPA1L1 in TM cells and aqueous humor samples obtained from patients with both POAG and cataracts (POAG group) and compared them to samples from patients with angle-closure glaucoma (ACG) and cataract or cataract-only (non-POAG group). Clinical characteristics of the 2 groups of patients are shown in Table 1. As illustrated in Figure 2A, the aqueous humor concentration of TGF-β2 in the non-POAG group (10 eyes from 10 patients) was 425.8 ± 62.3 pg/mL, whereas in the POAG group (8 eyes from 8 patients) it was notably higher at 1191.0 ± 271.9 pg/mL (*P* = 0.0077). The difference in TGF-β2 levels between POAG and non-POAG patients is similar to that previously reported (35–39). As shown in Figure 2B, and similarly to TGF-β2, the aqueous humor level of SIPA1L1 was 76.6 ± 1.5 ng/mL in the non-POAG eyes (*n* = 10) and significantly higher in the POAG eyes (96.8 ± 7.2 ng/mL, *n* = 8, *P* = 0.0075). We further investigated the potential relationship between IOP and TGF-β2, age and TGF-β2, IOP and SIPA1L1, as well as age and SIPA1L1. Our data indicate that within the control or the POAG group, TGF-β2 or SIPA1L1 did not statistically correlate with either IOP or age (Figure 2, C–F). Subsequently, we determined whether SIPA1L1 is elevated in HTM cells derived from POAG patients. Figure 2, G and H depicts that the concentration of SIPA1L1 in the POAG group (3 eyes from 3 patients) was significantly increased to 4.25 ± 0.23-fold (*P* < 0.0001) compared with the non-POAG groups (3 eyes from 3 patients). Our findings also confirmed that the IOP of POAG patients was significantly higher (*P* = 0.0026) than that of control (Supplemental Figure 1A; supplemental material available online with this article; https://doi.org/10.1172/jci.insight.174836DS1) (35–38), while age distributions of the 2 groups were similar (Supplemental Figure 1B). The increased level of SIPA1L1 in the aqueous humor and TM cells of POAG patients is intriguing. To validate whether TM cells secrete SIPA1L1, we utilized ELISA to evaluate SIPA1L1 concentrations in extracellular culture fluid samples obtained from control and TGF-β2 treatment groups. The results indicated a lower SIPA1L1 concentration in the control group (426.8 ± 72.5 ng/mL, *n* = 4) compared with the TGF-β2 group (674.6 ± 25.4 ng/mL, *n* = 6), with statistical significance (*P* = 0.0054, Supplemental Figure 2). These observations, together with the label-free quantitative proteomics study (Figure 1), suggest that TGF-β2 may directly or indirectly affect SIPA1L1 expression in the TM.

### Expression of SIPA1L1 was upregulated by TGF-β2 treatment in HTM cells.

**Figure 3:**
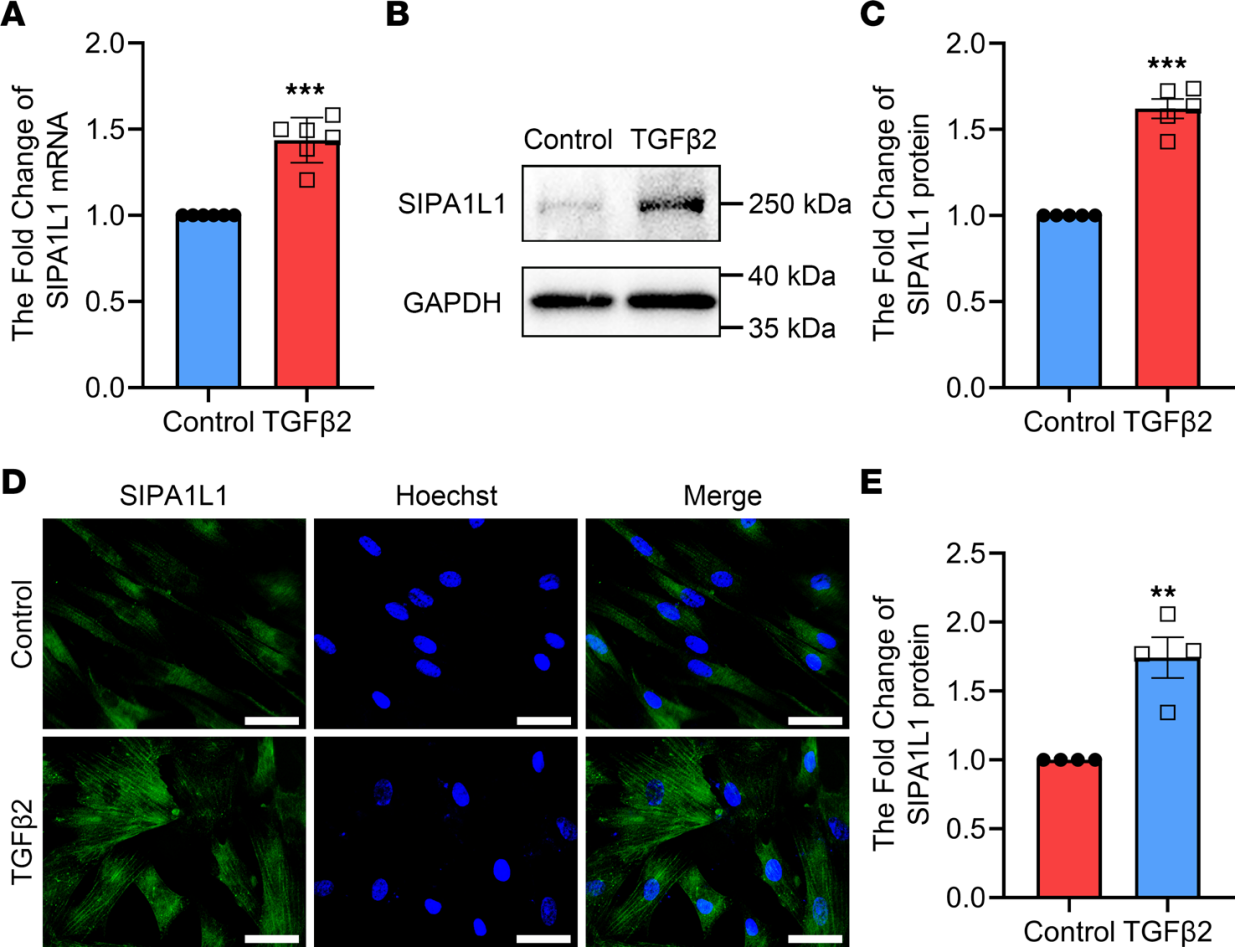
The expression of SIPA1L1 is upregulated by TGF-β2 induction in HTM cells. The HTM cells were treated with TGF-β2 (5 ng/mL) for 48 hours. (**A**) The change in SIPA1L1 mRNA level by RT-qPCR. (**B** and **C**) SIPA1L1 protein was analyzed by Western blotting. (**D** and **E**) SIPA1L1 protein was assessed by immunocytochemistry. Cells were counterstained with Hoechst 33342 nuclear dye. Each experiment used more than 2 individual HTM cell strains and data are shown as mean ± SEM (*n* = 5–6). **P < 0.01, ***P < 0.001 compared with control by unpaired, 2-tailed Student’s *t* test. Scale bars: 30 μm. Each symbol in graphs represents the data from each experiment.

We further evaluated the effect of TGF-β2 on SIPA1L1 expression in the HTM cells by real-time quantitative PCR (RT-qPCR), Western blotting, and immunocytochemistry. As shown in Figure 3A, after 48-hour treatment with 5 ng/mL TGF-β2, the SIPA1L1 mRNA level was significantly upregulated 1.44 ± 0.05-fold (*n* = 6, *P* < 0.0001) compared with the control group. Similarly, TGF-β2 treatment significantly increased the expression of SIPA1L1 protein to 1.62 ± 0.06-fold (*n* = 5, *P* < 0.0001 compared with the control group; Figure 3, B and C). These results were confirmed by immunocytochemistry (Figure 3, D and E). In the control cells, SIPA1L1 was minimally detected, while after TGF-β2 treatment, SIPA1L1 was observed throughout the cytoplasm of most HTM cells. These findings clearly demonstrate that SIPA1L1 level in the HTM cells is upregulated by TGF-β2.

### SIPA1L1 knockdown reduced the excessive expression of ECM in HTM cells.

**Figure 4:**
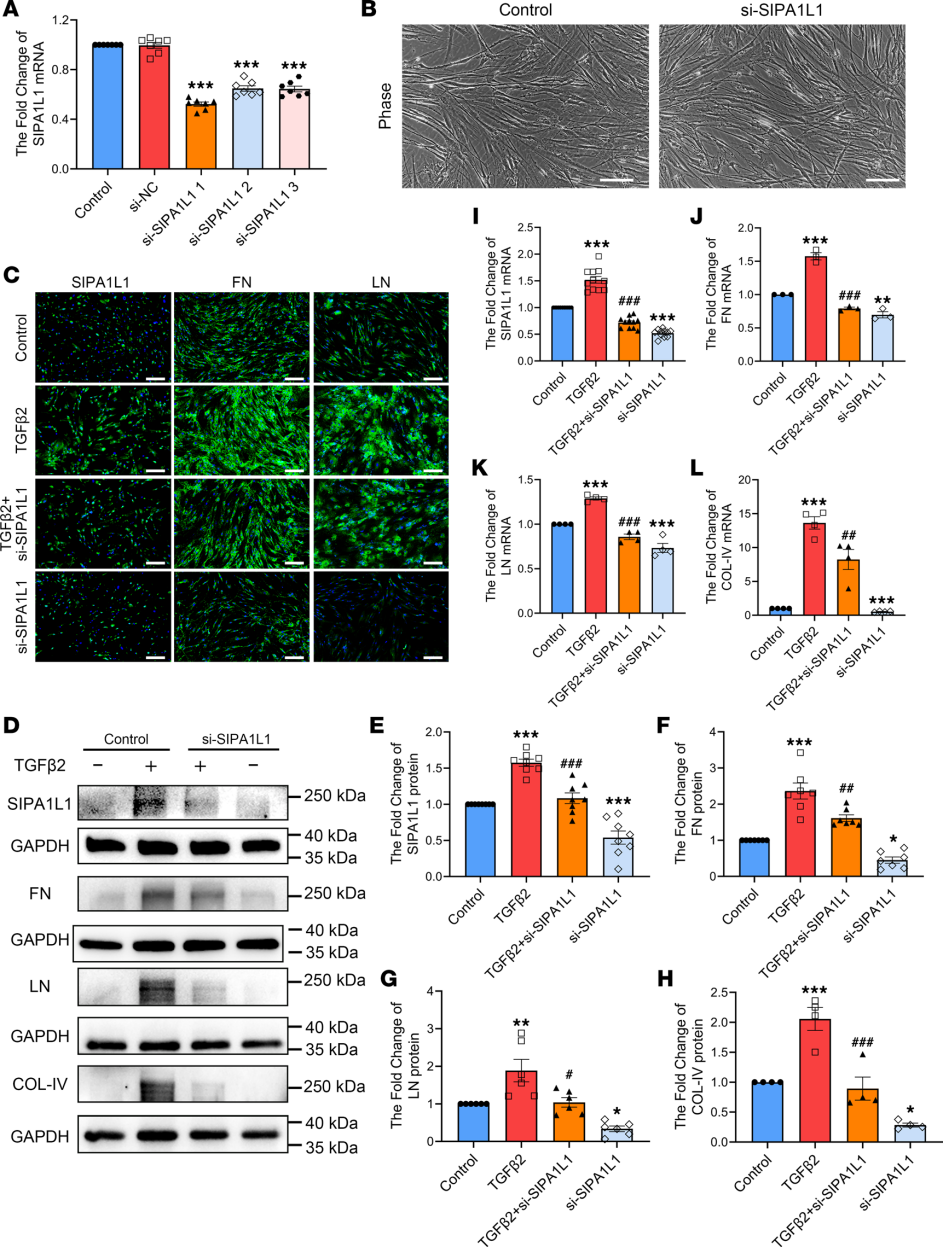
Knockdown of SIPA1L1 reduced expression of ECM in HTM cells. HTM cells were pretransfected with si-SIPA1L1 (20 μM) for 8 hours with or without TGF-β2 (5 ng/mL) treatment for 48 hours. (**A**) The knockdown efficacies of the 3 different tested si-SIPA1L1 were evaluated by RT-qPCR. Among the 3, si-SIPA1L1-1 was most efficacious and was used for the following knockdown studies. (**B**) After si-SIPA1L1 transfection, morphology of HTM cells was observed by microscopy and found to be normal. (**C**) The expression of SIPA1L1 and ECM proteins (FN, LN) were assessed by immunocytochemistry. (**D**–**H**) The levels of SIPA1L1 and 3 ECM proteins (FN, LN, COL-IV) were examined by Western blotting. (**I**–**L**) Levels of mRNA were evaluated by RT-qPCR. All studies used more than 4 individual HTM cell strains and data are expressed as mean ± SEM (*n* = 3–9). *P < 0.05, **P < 0.01, ***P < 0.001 compared with control; ^#^*P* < 0.05, ^##^*P* < 0.01, ^###^*P* < 0.001 compared with TGF-β2–treated group by 1-way ANOVA followed by Tukey’s multiple-comparison test. Scale bars: 100 μm (**B**) and 200 μm (**C**). Each symbol in graphs represents the data from each experiment.

Since TGF-β2 is capable of upregulating both SIPA1L1 and ECM proteins (fibronectin [FN], laminin [LN], and type IV collagen [COL-IV]), we further tested whether knockdown of SIPA1L1 affects the TGF-β2 effects on ECM expression. First, we evaluated the knockdown efficacies of 3 siRNAs against SIPA1L1, and found that, among the 3, si-SIPA1L1-1 was most efficacious, with a knockdown efficiency of 0.52 ± 0.02-fold (*n* = 7, *P* < 0.0001) compared with the control (Figure 4A). Hence, si-SIPA1L1-1 was used in the following knockdown studies. Transfection with si-SIPA1L1-1 did not affect the growth characteristics and morphology of HTM cells indicated by microscopic observations (Figure 4B).

Transfection with si-SIPA1L1 reduced the expression of FN and LN proteins in the HTM cells with or without TGF-β2 treatment. This effect was shown by immunocytochemistry (Figure 4C). Western blots also show similar results (Figure 4, D–H). Transfection of the HTM cells with si-SIPA1L1 significantly lowered the basal expression of SIPA1L1 (0.54 ± 0.09-fold, *n* = 8, *P* = 0.0001 compared with the control group), FN (0.45 ± 0.09-fold, *P* = 0.026), LN (0.34 ± 0.08-fold, *P* = 0.0476), and COL-IV (0.28 ± 0.03-fold, *P* = 0.0138). Treatment with TGF-β2 elevated the level of SIPA1L1 protein to 1.58 ± 0.05-fold (*P* < 0.0001) compared with the control group, along with FN (2.36 ± 0.22-fold, *P* < 0.0001), LN (1.89 ± 0.30-fold, *P* = 0.0072), and COL-IV (2.06 ± 0.19-fold, *P* = 0.0007), which were also significantly increased. Knockdown of SIPA1L1 decreased the effects of TGF-β2, such that it brought the SIPA1L1 level back to the untreated baseline level (1.08 ± 0.07-fold, *P* < 0.0001 compared with the control group). The levels of FN, LN, and COL-IV were also significantly decreased compared with the respective TGF-β2–treated groups (Figure 4, D–H). In addition to protein levels, similar results were also observed in mRNA changes. Figure 4I shows that the level of SIPA1L1 mRNA was elevated to 1.52 ± 0.06-fold (*P* < 0.0001 compared with the control group) by TGF-β2. Similarly, the levels of FN, LN, and COL-IV mRNA were all increased after TGF-β2 treatment (Figure 4, J–L). Knockdown of SIPA1L1 with siRNA lowered the expression of SIPA1L1 to 0.72 ± 0.03-fold (*P* < 0.0001) compared with the control group, and the levels of FN, LN, and COL-IV were also decreased (Figure 4, I–L). These results indicate that SIPA1L1 plays an important role in the TGF-β2–induced expression of ECM in the HTM cells.

### SIPA1L1 knockdown increased phagocytosis through regulating cytoskeletal actin in HTM cells.

**Figure 5:**
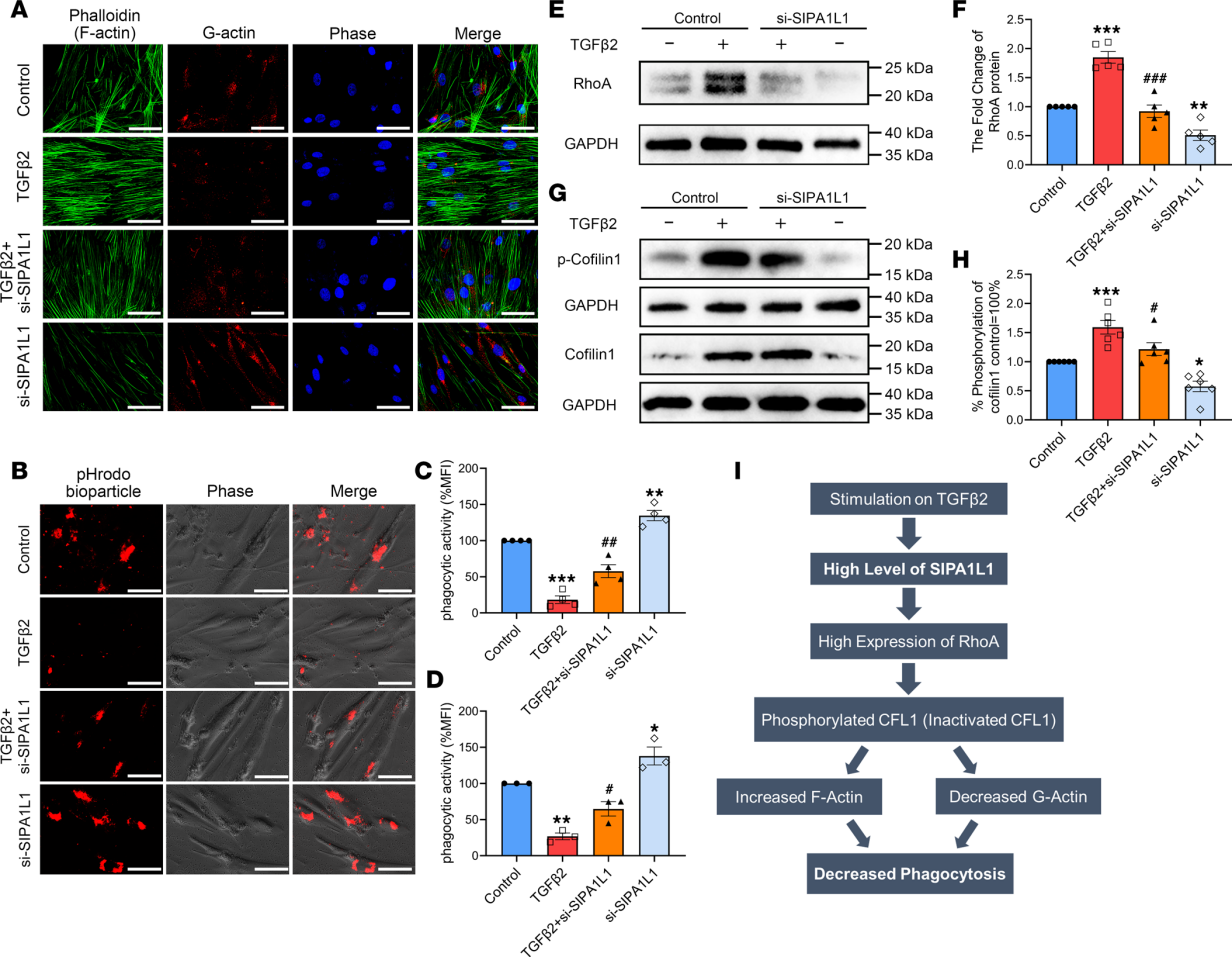
Knockdown SIPA1L1 promoted phagocytosis in HTM cells. HTM cells were transfected with si-SIPA1L1 (20 μM) for 8 hours with or without TGF-β2 (5 ng/mL) for 48 hours. (**A**) Expression of cytoskeletal proteins (F-actin indicated by phalloidin staining, green; G-actin, red) is shown with fluorescence micrographs. (**B**) Phagocytic activity was evaluated by incubating pHrodo Red bioparticles with the cells for 3 hours. (**C** and **D**) Red fluorescence intensity of pHrodo bioparticles was quantified by ImageJ and FACS. (**E**–**H**) Western blots were used to assess RhoA protein level and phosphorylated CFL1 protein level. (**I**) The proposed SIPA1L1 signaling pathway associated with phagocytosis is shown schematically. All results used more than 3 individual HTM cell strains and data are expressed as mean ± SEM (*n* = 4–6). *P < 0.05, **P < 0.01, ***P < 0.001 compared with control; ^#^*P* < 0.05, ^##^*P* < 0.01, ^###^*P* < 0.001 compared with TGF-β2–treated group by 1-way ANOVA followed by Tukey’s multiple-comparison test. Scale bars: 30 μm. Each symbol in graphs represents the data from each experiment.

Phagocytosis is an important function of TM cells, which can remove debris from the TM region to minimize outflow obstruction (28, 40). Meanwhile, SIPA1L1 is closely associated with actin cytoskeletal organization by affecting RhoA. To further explore the potential cellular mechanisms affected by SIPA1L1 in TM functions, we evaluated the actin cytoskeleton in HTM cells. We found that formation of F-actin, as indicated by phalloidin staining (Figure 5A), was markedly increased by TGF-β2 and reduced by si-SIPA1L1, while G-actin staining (red) had an opposite tendency; it was decreased by TGF-β2 and increased by si-SIPA1L1 (Figure 5A). These results show that SIPA1L1 is required for actin remodeling, which is an important step of phagocytosis in the TM cells.

To directly evaluate phagocytic activity of the HTM cells, the cells were incubated with pHrodo bioparticles and phagocytosis was observed by its emission of red fluorescence when the particles were phagocytosed and transported to the lysosome. As shown in Figure 5, B–D, HTM cells had a baseline level of phagocytic activity, which was significantly suppressed by TGF-β2 (26.8% ± 4.6%, *n* = 3, *P* = 0.0011 compared with the control group). Transfection of HTM cells with si-SIPA1L1 partially reversed the suppressive effect of TGF-β2, such that the phagocytic activity returned to 64.8% ± 9.9% (*P* = 0.0469 compared with the control group). Interestingly, si-SIPA1L1 alone increased the cell’s phagocytic activity to 137.9% ± 12.4% (*P* = 0.0474 compared with the control group), which indicates that knockdown of SIPA1L1 alone could promote phagocytosis in HTM cells (Figure 5, B–D). Subsequently, we confirmed the protein levels of a signaling pathway relevant to phagocytosis. As shown in Figure 5, E–H, after TGF-β2 treatment, RhoA was upregulated (1.85 ± 0.10-fold, *P* < 0.0001) and the percentage of phosphorylated CFL1 (p-CFL1/CFL1) was also increased (59.2% ± 11.6% increase, *P* = 0.001) compared with the control group. Knockdown of SIPA1L1 ameliorated the TGF-β2–increased expression of RhoA (0.92 ± 0.11-fold, *P* < 0.0001 compared with the control group) and percentage of p-CFL1/CFL1 was reduced to 37.7% ± 0.5% (*P* = 0.04 compared with the TGF-β2 group). Meanwhile, si-SIPA1L1 alone decreased the expression of RhoA (0.51 ± 0.09-fold, *P* = 0.0046) and the ratio of p-CFL1/CFL1 (42.3% ± 8.9% reduction, *P* = 0.0188) compared with the control group (Figure 5, E–H).

These results suggest that SIPA1L1 can induce phosphorylation of CFL1 through upregulation of RhoA, and p-CFL1 increases formation of F-actin, which reduces phagocytosis. A schematic representation of this hypothesis is shown in Figure 5I. The results also indicate that removing SIPA1L1 alone increases phagocytosis of HTM cells by regulating cytoskeletally relevant proteins such as RhoA and CFL1, which may help to reduce the accumulation of ECM in the TM.

### SIPA1L1 overexpression led to upregulated ECM expression and decreased phagocytic activity by modulating cytoskeletal actin dynamics in HTM cells.

**Figure 6:**
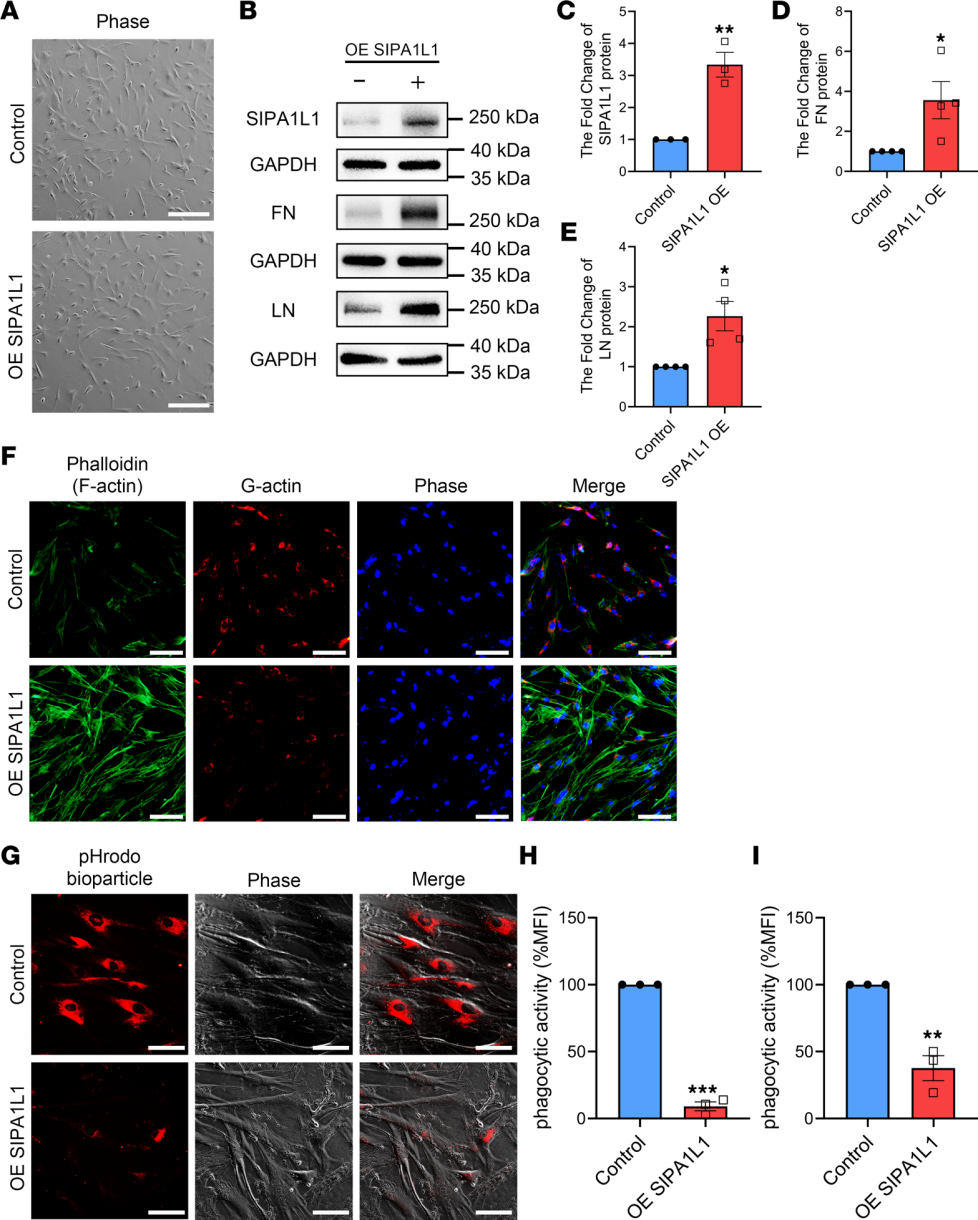
The overexpression of SIPA1L1 stimulated ECM expression and attenuated phagocytic activity in HTM cells. HTM cells were transfected with OE SIPA1L1 plasmid (1 ng/μL) for 8 hours. (**A**) The morphology of the transfected HTM cells was observed under a microscope and found to be normal. (**B**–**E**) Western blot analysis was used to examine the levels of SIPA1L1 and ECM proteins (FN, LN). (**F**) Expression of cytoskeletal proteins (F-actin indicated by phalloidin staining, green; G-actin, red) were visualized using fluorescence micrographs. (**G**) Phagocytic activity of the cells was evaluated by incubating pHrodo Red bioparticles with the cells for 3 hours. (**H** and **I**) The red fluorescence intensity of the bioparticles was quantified using ImageJ and FACS. All results used more than 3 individual HTM cell strains and data are expressed as mean ± SEM (*n* = 3–4). *P < 0.05, **P < 0.01, ***P < 0.001 compared with control by unpaired, 2-tailed Student’s *t* test. Scale bars: 400 μm (**A** and **G**) and 100 μm (**F**). Each symbol in graphs represents the data from each experiment.

To ascertain the role of SIPA1L1 in ECM accumulation without TGF-β2, we conducted additional experiments to investigate the impact of SIPA1L1 overexpression on ECM expression. Firstly, we observed that transfection with a plasmid overexpressing SIPA1L1 (OE SIPA1L1) did not alter the growth characteristics or morphology of HTM cells, as evidenced by microscopic observations (Figure 6A). Subsequently, as depicted in Figure 6, B–E, we observed a significant increase in SIPA1L1 overexpression to 3.34 ± 0.28-fold (*n* = 3, *P* = 0.0037) compared with the control group. This overexpression was accompanied by the upregulation of FN (3.56 ± 0.94-fold, *n* = 4, *P* = 0.0338) and LN (2.27 ± 0.37-fold, *n* = 4, *P* = 0.0136). These findings suggest that elevated SIPA1L1 expression alone is adequate to enhance the accumulation of ECM in HTM cells.

In order to investigate the role of SIPA1L1 in cellular phagocytosis independently of TGF-β2, we conducted experiments to evaluate the impact of SIPA1L1 overexpression on intracellular actin cytoskeletal organization and phagocytic activity. As illustrated in Figure 6F, the presence of F-actin (green) was significantly enhanced upon SIPA1L1 overexpression, while G-actin (red) was reduced. Moreover, in Figure 6, G–I, the phagocytic activity of HTM cells was notably inhibited following transfection with the OE SIPA1L1 plasmid (37.6% ± 9.3%, *n* = 3, *P* = 0.0026 compared with the control group). These findings suggest that heightened SIPA1L1 expression alone can diminish cellular phagocytosis by promoting F-actin accumulation.

## Discussion

Our laboratory has been interested in the pathogenesis of glaucoma and its treatment. We have been evaluating cellular signaling pathways and mechanisms involved in the excessive accumulation of ECM in HTM cells as well as in mouse TM tissue with TGF-β2 treatment (41–43). TGF-β2, a member of the TGF superfamily, binds to TGF-β receptor 2 (TGFBR2) and subsequently recruits and activates TGFBR1, initiating the TGF-β signaling pathway. Within TM cells, TGF-β2 regulates the expression of ECM protein genes through the Smad signaling pathway (44). Upon activation, TGF-β2 induces the phosphorylation and nuclear translocation of Smad proteins, thereby facilitating the transcription of specific genes encoding ECM-related proteins. Furthermore, TGF-β2 can modulate the expression of ECM proteins in TM cells via non-Smad signaling pathways, including the Rho GTPase, mitogen-activated protein kinase (MAPK), and phosphoinositide 3-kinase (PI3K) pathways, among others (12, 45, 46). Activation of TGF-β2 stimulates these signaling pathways, resulting in the regulation of ECM protein synthesis and secretion. In the current study, we found that SIPA1L1 was elevated in the aqueous humor of POAG patients and in TGF-β2–treated HTM cells. This protein not only promotes the production of ECM proteins in these cells, but also inhibits the degradation of ECM proteins by inhibiting phagocytosis.

SIPA1L1 has been studied in other tissues. It was reported that SIPA1L1 can mediate the endocytosis of AQP2 in epithelial cells of the kidney collecting duct by activating its RapGAP domain (22). Moreover, casein kinase Iε can phosphorylate SIPA1L1 and promote its degradation, alleviating SIPA1L1-mediated inhibition of Rap1 to regulate gastrulation (21). In regard to fibrosis, SIPA1L1 is expected to be an early detection indicator for liver fibrosis caused by carbon tetrachloride (32). However, the role of SIPA1L1 in HTM cells is unknown, especially whether SIPA1L1 affects ECM accumulation in HTM cells.

Using label-free quantitative proteomics, we evaluated the expression patterns of up- and downregulated proteins through heatmaps. Notably, ECM-associated proteins (FN, LN, COL-IV) exhibited increased expression in the TGF-β2 group, confirming the effects of TGF-β2 in TM cells and validating the reliability of mass spectrometry (MS) data. Integrins, integral transmembrane heterodimeric cell surface receptors comprising α and β subunits, have emerged as pivotal regulators of phagocytosis across various cell types (47, 48). Concurrently, we observed an upregulation in integrin isoforms following TGF-β2 treatment, indicating TGF-β2’s ability to modulate phagocytosis in TM cells. Importantly, our findings underscored FN’s dual role in ECM-receptor interactions and the regulation of the actin cytoskeleton pathways. Existing reports suggest that FN collaborates with Toll-like receptors 2 and 4 (TLR2 and TLR4) to enhance macrophage phagocytosis of bacteria by binding to integrin β1 (49). We posited a similar function for FN in TM cells, and future experiments will elucidate how ECM constituents enhance cellular phagocytosis. In our study, we used label-free quantitative proteomics to comprehensively describe the ECM accumulation pathways (ECM-receptor interaction and regulation of actin cytoskeleton) and revealed that SIPA1L1 was one of the proteins prominently elevated upon TGF-β2 treatment (Figure 1).

Our prior investigation has demonstrated a significant elevation in the concentration of TGF-β2 within the aqueous humor of patients diagnosed with POAG (35). In the present study, we have extended our findings by demonstrating a significant increase in the levels of both SIPA1L1 and TGF-β2 in the aqueous humor and TM cells of POAG patients compared with non-POAG patients (Figure 2). These results suggest a potential association between SIPA1L1 and TGF-β2 in the pathogenesis of POAG. Subsequent investigations revealed that the upregulation of SIPA1L1 was induced by TGF-β2. Furthermore, we observed a downregulation in the levels of ECM components (FN, LN, COL-IV) upon knocking down SIPA1L1 in HTM cells (Figures 3 and 4). These findings suggest that TGF-β2 acts as an upstream signaling pathway, promoting ECM production through SIPA1L1. Importantly, the exclusive downregulation of SIPA1L1 resulted in a notable decrease in the basal expression of ECM proteins and mRNA. These intriguing findings suggest that SIPA1L1 may have the capacity to influence ECM accumulation independently of TGF-β2. Thereby, SIPA1L1 may be involved in TGF-β2–mediated induction, as well as non–TGF-β2 pathway–induced ECM accumulation.

In addition to ECM production, SIPA1L1 appears to affect TM cell phagocytosis. Phagocytosis is a cellular process that engulfs macromolecules and large energy-rich particles (typically larger than 0.5 μm) into cells, mediated by the remodeling of the actin cytoskeleton (50, 51). It can be found in many types of cells such as macrophages, neutrophils, monocytes, dendritic cells, osteoclasts, and especially TM cells (48, 52–54). The phagocytosed particles are internalized into the phagosome, and then the phagosomes are recognized and further fused with lysosomes to mature into phagolysosomes, in which the ingested materials are degraded (50, 52, 55–58). Phagocytosis is one of the critical biological functions of TM cells, which is closely related to ECM remodeling and regulation of IOP (59). It has been shown that, in TM cells, phagocytosis degrades ECM proteins and maintains the trabecular aqueous humor outflow facility free of cellular debris (40, 47, 60, 61). Phagocytotic activity depends on cytoskeletal organization and the transformation of F-actin to G-actin, which integrin and RhoA are known to regulate. RhoA, by modifying the activity of CFL1, regulates the depolymerization of F-actin to form G-actin (25, 31). Thus, RhoA activation is capable of reducing TM cellular phagocytosis, and Rho-associated protein kinase inhibition can increase phagocytotic activity in TM cells (28, 29, 62). SIPA1L1 can regulate actin cytoskeletal configuration via RhoA. However, whether SIPA1L1 is directly involved in phagocytosis has not been previously reported.

In this study, we showed that TGF-β2 reduced phagocytosis of HTM cells, and this effect was ameliorated by knocking down SIPA1L1. These changes were associated with changes in RhoA and CFL1. For instance, TGF-β2 increased the levels of both RhoA and phosphorylated CFL1, and both were partially reversed by SIPA1L1 knockdown (Figure 5). Interestingly, downregulation of SIPA1L1 alone also promoted F-actin depolymerization and enhanced phagocytic activity in HTM cells.

These results suggest that knocking down SIPA1L1 not only decreases ECM gene production, preventing ECM proteins from being produced, but also stimulates phagocytic activity in TM cells, enhancing their ability to remove ECM proteins that have previously accumulated. Thus, reduction of SIPA1L1 level can reverse the glaucomatous effects of TGF-β2 in the TM.

To investigate the independent impact of SIPA1L1 on the morphology and functionality of HTM cells while excluding the influence of TGF-β2, we focused on overexpressing SIPA1L1. Our experimental results demonstrated that the overexpression of SIPA1L1 led to the upregulation of ECM protein expression and a reduction in cellular phagocytic activity compared with the control group. Notably, the morphology of HTM cells remained unaffected throughout the experiment (Figure 6). These findings underscore the pivotal role of SIPA1L1 in modulating ECM expression and phagocytosis within HTM cells.

At this time, the exact signaling molecules and steps involved in the effects of SIPA1L1 and its expression are not fully elucidated. According to previously published reports, SIPA1L1 can inhibit activation of Rap1 by its RapGAP domain (21) to activate Arf and Rho GAP adapter protein 3 (Arap3), which prevents Arap3 from being able to inactivate RhoA through its RhoGAP domain (63–65). Our experimental results elucidated that the targeted suppression of SIPA1L1 resulted in a discernible decrease in RhoA expression. This observation indicates the inherent capacity of SIPA1L1 to actively modulate and promote the levels of RhoA. Simultaneously, analysis through the KEGG pathway database corroborated the identification of SIPA1L1 within the same signaling pathway. SIPA1L1, recognized as HPV E6-targeted protein 1 (E6TP1), was implicated in facilitating the hydrolysis of Rap1GTP (activated state) to Rap1GDP (inactivated state). This process, in turn, modulates F-actin through the Arap3/RhoA pathway (Supplemental Figure 3). Therefore, we hypothesized that SIPA1L1 could regulate the Rap1/Arap3 pathway in TM cells to activate RhoA, and the specific mechanism will be addressed in future studies.

According to our findings, SIPA1L1 was present in higher concentrations in the aqueous humor and TM cells of POAG patients, and TGF-β2 did increase the release of SIPA1L1 into TM cell extracellular culture medium. However, it is uncertain whether SIPA1L1 has extracellular functions, how it is secreted into extracellular fluids, and whether TGF-β2 affects the process. SIPA1L1 is predominantly considered to regulate the cytoskeleton through Rap1 within the cell. As a GTPase, SIPA1L1 has only been reported to function intracellularly. Upon comparing SIPA1L1’s protein sequence, we observed the absence of a signal peptide common in secretory proteins. We speculate that it may be secreted extracellularly through a non-classical protein secretion pathway. We will continue to investigate this intriguing phenomenon, unraveling SIPA1L1’s extracellular transport pathways and related functions and examining its potential involvement in the accumulation of ECM proteins in the TM and elevated IOP.

Nonetheless, based on our findings and those published by others, we propose a potential mechanism for the dual effects of SIPA1L1 on ECM production and its degradation. Future studies pinpointing the exact molecular and cellular mechanisms of SIPA1L1 and its interaction with other modulating factors will be very important in the evaluation of this interesting protein and its potential clinical usefulness in the treatment of glaucoma.

## Methods

### Sex as a biological variable.

Sex was not considered as a biological variable. In the early stages of this study, we analyzed TM cell strains and aqueous humor samples from male and female patients, revealing comparable findings across both sexes.

### Cell culture and treatment.

Primary HTM cells were derived from HTM tissues during trabeculectomy procedures for ACG in accordance with the principles of the Declaration of Helsinki. Our TM cell strains were derived from a total of 14 patients. The specifics of HTM donors are presented in Supplemental Table 1. The HTM cell culture and identification have been described by us previously (41). Briefly, they were cultured at 37°C and 5% CO_2_ in Dulbecco’s modified Eagle’s medium (Gibco) supplemented with glutamine, penicillin, streptomycin, and 10% fetal bovine serum (Invitrogen/Gibco). Cultured cells of the third to ninth passages were used in the present studies. All treatments were performed at approximately 80% cell confluence. The cells were serum starved with Eagle’s medium for 24 hours before treatment with TGF-β2 (5 ng/mL, MedChemExpress) for 48 hours with fresh medium.

### Label-free quantitative proteomics technology.

Samples were prepared from cultured HTM cells. The protein extraction was performed by the SDT lysis method. Briefly, the appropriate amount of SDT lysis buffer was added to the samples, which was sonicated and then incubated in boiling water for 15 minutes. Next, the samples were centrifuged and the supernatant was collected and stored at –80°C. Protein quantification was performed using the BCA method and the initial protein concentration in control and TGF-β2 groups was 5.2 μg/μL and 4.6 μg/μL, respectively. The protein from each sample (20 μg) was added to 6× loading buffer and then resolved in 12% SDS-PAGE. Finally, the gel was stained by Coomassie Brilliant Blue. Filter-aided sample preparation (FASP) for MS-based proteomic analysis was described by Wisniewski et al. (66). In brief, dithiothreitol (DTT) was added to each protein sample (80 μg) to 100 mM final concentration. The samples were mixed with urea (8 M) to dissociate detergent micelles and protein detergent complexes. The detergent, DTT, and other low-molecular-weight components were removed by utrafiltration via centrifugation. Subsequently, iodoacetamide (100 mM) was added to samples to carboxyamidomethylate with thiols. The samples were washed twice with 8 M urea and 40 mM NH_4_HCO_3_ solutions. The samples were subsequently mixed with trypsin buffer (trypsin in 40 mM NH_4_HCO_3_) and centrifuged. Finally, these protein suspensions were digested with endoproteinase and the resulting peptides were collected as a filtrate. The combined filtrates were desalted in MILI-SPE Extraction disk C_18_ cartridges. The peptides were analyzed by UV light spectral density at 280 nm.

The Easy nLC system was used to resolve samples followed by Q Exactive Plus to analyze them (both Thermo Fisher Scientific). The resolved samples were processed by an analytical column (Acclaim PepMap RSLC 50 μm × 15 cm, NanoViper; 164943, Thermo Fisher Scientific) at a flow rate of 300 nL/min. The mobile phase contained 0.1% formic acid aqueous solution (phase A) and 0.1% acetonitrile aqueous solution (phase B). The samples were separated in a 2-hour gradient as follows: 100% phase A was unaltered the entire time, 3% phase B from 0 to 5 minutes, 3% to 28% phase B from 5 to 95 minutes, 28% to 38% phase B from 95 to 110 minutes, 38% to 100% phase B from 110 to 115 minutes, and 100% phase B was stabilized at 115–120 minutes. Data-related acquisitions with a first-class MS resolution of 70,000 (at 200 *m*/*z*), ACG target of 3 × 10^6^, and first-class maximum IT for 50 ms were performed. The *m*/*z* ratio of polypeptide and polypeptide fragments was acquired by MS2 scan after full scan, MS2 activation type for HCD, isolation window of 2 *m*/*z*, second-class MS resolution of 17,500, microscans of 1, second-class maximum IT for 45 ms, and normalized collision energy of 27 eV.

MaxQuant software (version 1.5.5.1) was used for database searches, and the Label-Free Quantitation algorithm was utilized for quantitative analysis (67); we used a comprehensive protein database: Uniprot_HomoSapiens_20386_20180905. The number of maximum missed cleavages was less than 2, and the minimum criterion for unique peptides required for protein identification was 1 or greater. Bioinformatic analysis was performed using the OmicStudio tools (https://www.omicstudio.cn/tool/), and the clustering heatmap and KEGG pathway enrichment were drawn from the websites (68).

### ELISA.

Soluble SIPA1L1 and TGF-β2 proteins were quantified in aqueous humor using human SIPA1L1 and TGF-β2 ELISA kits (Fine Biotech). Aqueous humor samples from POAG patients with cataracts were compared to samples from cataract-only eyes (controls). Patient information is listed in Table 1. Aqueous humor was collected during cataract surgery and stored at –80°C until analysis. All samples were diluted 40-fold in sample dilution buffer and analyzed according to the manufacturer’s protocol. All samples were assayed in the same instrument under the same conditions.

### SIPA1L1 siRNA and OE plasmid transfection.

The SIPA1L1 siRNA (si-SIPA1L1) was designed and synthesized by GenePharma. The plasmid overexpressing SIPA1L1 (OE SIPA1L1) was custom designed and synthesized by Bioscience. The siRNA sequences are shown in Supplemental Table 2 and the *Homo* *sapiens* SIPA1L1 gene sequence was obtained from the National Center for Biotechnology Information (NCBI). After 24-hour serum starvation, HTM cells were transfected with si-SIPA1L1-1, -2, -3 (20 μM), or plasmid OE SIPA1L1 (1 ng/μL) using Lipofectamine 3000 reagent (Thermo Fisher Scientific) according to the manufacturer’s protocols. Briefly, in 6-well plates, Lipofectamine 3000 reagent (5 μL) and siRNA or plasmid (5 μL) were dissolved in 250 μL of single culture medium for 5 minutes, mixed and incubated for 15 minutes, and then added to the cells containing 1.5 mL of medium. The total time of transfection was approximately 8 hours.

### Phagocytosis of pHrodo Red bioparticles.

The pHrodo Red bioparticles (Thermo Fisher Scientific), a *Staphylococcus aureus* bioparticle conjugate, have low fluorescence at neutral or high pH, but have high fluorescence at low pH, such as in lysosomes. The HTM cell phagocytosis was evaluated according to the manufacturer’s instructions. pHrodo bioparticles were resuspended using Live Cell Imaging Solution (pH 7.4, Thermo Fisher Scientific) in a centrifuge tube and sonicated for 5 minutes, until all the bioparticles were homogeneously dispersed. Next, the cell culture medium of HTM cells in a 24-well plate was quickly replaced with 250 μL of the prepared pHrodo bioparticle suspension. Subsequently, the 24-well plate was incubated at 37°C and 5% CO_2_ for 3 hours to allow phagocytosis and acidification in lysosomes. The extracellular bioparticle suspension was then removed and fresh Live Cell Imaging Solution was added to the cells. Images under a fluorescence microscope (BX51, Olympus Optical Co. Ltd.) were recorded.

### FACS analysis.

After the HTM cells were incubated with the pHrodo Red bioparticles at 37°C and 5% CO_2_ for 3 hours in a 24-well plate, the cells were washed with PBS 3 times. Subsequently, the cells were detached using a 0.25% trypsin solution for 1 minute and then resuspended in complete medium containing 2% serum. The cell suspension was then collected, and the fluorescence intensity of the pHrodo bioparticles was analyzed using the BD FACS DIVA System (BD Fortessa). The phagocytic activity was assessed by quantifying the geometric mean fluorescence intensity (MFI) of phagocytosed bioparticles. The percentage of phagocytic activity relative to the control was calculated by determining the ratio of the MFI for treated samples to that of untreated samples, multiplied by 100.

### RT-qPCR.

Total RNA was extracted from cultured HTM cells using Trizol (Takara). The subsequent synthesis of cDNA was performed using the PrimeScript RT Reagent Kit (Takara) according to the manufacturer’s instructions. RT-qPCR was performed using SYBR Green Master Mix (Qiagen) to evaluate mRNA levels. Human glyceraldehyde-3-phosphate dehydrogenase (GAPDH) and mouse β-actin were used as standards. Primer sequences used for the qPCR reactions are shown in Supplemental Table 3.

### Western blot analysis.

Total cellular proteins from HTM cells were isolated using RIPA lysis buffer (CWBIO) with protease and phosphatase inhibitor cocktails (Cell Signaling Technology [CST]). The concentrations of proteins were measured by Pierce BCA Protein Assay Kit (Thermo Fisher Scientific), according to the manufacturer’s instructions. All samples were denatured at 100°C for 10 minutes, resolved by SDS-PAGE, and all proteins were transferred to a polyvinylidene difluoride (PVDF) membrane, which was blocked with 5% BSA for 1 hour and then incubated overnight at 4°C with the appropriate primary antibody. On the next day, the membrane was washed and incubated with horseradish peroxidase–conjugated anti–rabbit IgG (1:5000; 14708, CST) for 1 hour at room temperature. Finally, the membranes were treated with an Omni-ECL Femto Light Chemiluminescence Kit (Epizyme Biotech) and scanned using an ECL Plus Western Blot Detection Kit (Tanon), and then quantified by gray value with ImageJ software (NIH). The primary antibodies included rabbit polyclonal anti-SIPA1L1 antibody (1:500; 25086-1-AP, Proteintech), rabbit polyclonal anti-FN antibody (1:5000; ab2413, Abcam), rabbit polyclonal anti-LN antibody (1:5000; ab11575, Abcam), rabbit polyclonal anti–COL-IV antibody (1:1000; ab6586, Abcam), rabbit polyclonal anti-RhoA antibody (1:1000; ab187027, Abcam), rabbit polyclonal anti-CFL1 antibody (1:1000; AF6232, Affinity Biosciences), rabbit polyclonal anti–phospho-CFL1 antibody (1:1000; AF3232, Affinity Biosciences), and rabbit polyclonal anti-GAPDH (1:5000; 5174, CST). GAPDH was used as an internal control and to normalize protein signals. The protein density was analyzed by ImageJ software.

### Immunocytochemistry.

HTM cells were cultured on coverslips in 24-well plates. After the indicated treatment was completed, cells were fixed and permeabilized by 4% paraformaldehyde with 0.5% Triton X-100 (Thermo Fisher Scientific) at 4°C for 30 minutes and then blocked in 10% goat serum at 4°C for 1 hour. Subsequently, the cells were incubated in primary antibodies at 4°C overnight, including rabbit polyclonal anti-SIPA1L1 antibody (1:200; Proteintech), rabbit polyclonal anti-FN antibody (1:200; Abcam), rabbit polyclonal anti-LN antibody (1:200; Abcam), rabbit polyclonal anti–COL-IV antibody (1:200; Abcam), or rabbit polyclonal anti–actin α1 antibody for G-actin expression levels (1:200; Affinity). After washing in PBS 3 times, the Alexa Fluor 488–conjugated or Alexa Fluor 594–conjugated anti-rabbit antibody as the secondary antibody (1:1000; Invitrogen) was used to incubate the HTM cells at room temperature protected from light for 1 hour. Afterward, the slides were stained with Hoechst 33258 (1:5000; Yeason) at 4°C for 15 minutes and/or incubated in phalloidin (1:1000; Yeason) at room temperature for 30 minutes. Finally, the fluorescence images were obtained under a fluorescence microscope (Nikon).

### Statistics.

Data are expressed as mean ± standard error of the mean (SEM). Data were analyzed by unpaired, 2-tailed Student’s *t* test for comparisons between 2 groups and 1-way analysis of variance (ANOVA) followed by Tukey’s test for comparisons among 3 or more groups. A *P* value of less than 0.05 was considered to be statistically significant. In all experiments, *n* was 3 or greater.

### Study approval.

All experiments were performed in Shanghai Key Laboratory of Orbital Diseases and Ocular Oncology. The study complied with the Declaration of Helsinki. The TM and aqueous humor samples from patients were obtained from the Ninth People’s Hospital, Shanghai Jiao Tong University School of Medicine following approval by the institutional research ethics board (No. SH9H-2019-TK204-1).

### Data availability.

Label-free quantitative proteomics data have been deposited in UniProt (https://www.uniprot.org/; Uniprot_HomoSapiens_20386_20180905). All raw data values used in the graphs are available in the Supporting Data Values file. Upon reasonable request, the lead corresponding author will provide access to the data that underpin the study’s conclusions.

## Author contributions

CX, MH, and DS performed the experiments and collected clinical samples. CX contributed to analyzing the data and writing the manuscript. TG, HS, JW, and SZ designed research studies. HS, MH, and CX modified the manuscript. TG provided human samples. TG and HS supervised the project. Co–first authors were ordered by experimental and draft writing contribution. All authors read and approved the manuscript.

## Supplementary Material

Supplemental data

Unedited blot and gel images

Supporting data values

## Figures and Tables

**Table 1:**
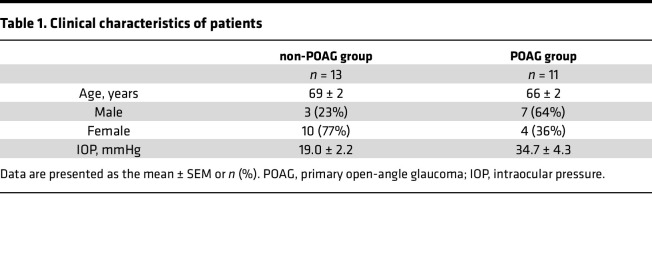
Clinical characteristics of patients

## Version 1 10/03/2024

In-Press Preview

## Version 2 11/22/2024

Electronic publication
