# Supplementary material for: Effects of SIPA1L1 on trabecular meshwork extracellular matrix protein accumulation and cellular phagocytosis in POAG
Source: JCI Insight. 2024 Nov 22;9(22):e174836. doi: 10.1172/jci.insight.174836 (PMC11601898; doi:10.1172/jci.insight.174836)
Supplement: Supplemental data [file jciinsight-9-174836-s053.pdf]

Supporting information

Effects of SIPA1L1 on trabecular meshwork extracellular matrix protein accumulation and cellular phagocytosis in POAG

Chenyu Xu[1-3], Jiahong Wei[4], Dan Song[1-3, 5], Siyu Zhao[5], Mingmin Hou[3], Yuchen Fan[6], Li Guo[7-8], Hao Sun[1-2] and Tao Guo[1-2]

[1]Department of Ophthalmology, Ninth People's Hospital, Shanghai Jiao Tong University School of Medicine, Shanghai, China
[2]Shanghai Key Laboratory of Orbital Diseases and Ocular Oncology, Shanghai, China
[3]Bengbu Medical College, Bengbu, China
[4]Department of Ophthalmology, Songjiang Hospital, Shanghai Jiao Tong University School of Medicine, Shanghai, China
[5]Department of Ophthalmology, Tongji Hospital, Tongji University School of Medicine, Shanghai, China
[6]Department of Ophthalmology, First Affiliated Hospital of Bengbu Medical College, Bengbu, China
[7]Lu'an Hospital Affiliated to Anhui Medical University, Lu'an, China
[8]Lu'an People's Hospital, Lu'an, China

CX, JW, DS and SZ contributed equally to this work.

Address correspondence to: Tao Guo, Department of Ophthalmology, Ninth People's Hospital, Shanghai Jiao Tong University School of Medicine, 639 Zhizaoju Road, Huangpu, Shanghai 200011, China. Phone: 86-13764923689. Email: guotao9@hotmail.com. Or to Hao Sun, Department of Ophthalmology, Ninth People's Hospital, Shanghai Jiao Tong University School of Medicine, 639 Zhizaoju Road, Huangpu, Shanghai 200011, China. Phone: 86-13671924498. Email: Sunhao6666@126.com.

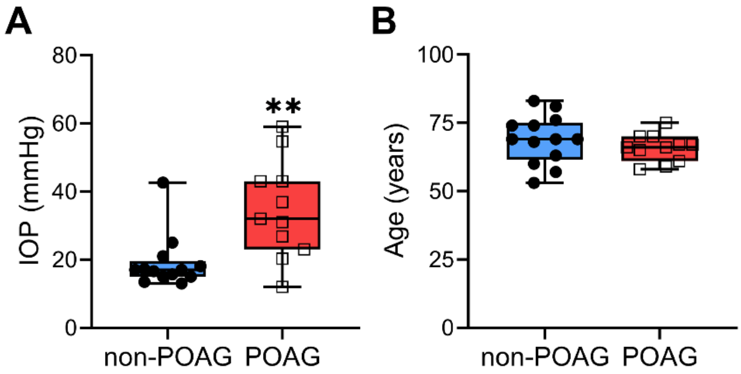

Figure S1. The correlation between IOP and age in patients. (A, B) The differences in IOP and age between non-POAG and POAG patients are shown. Data are expressed as mean ± SEM (n = 11-13). **: $P < 0.01$ compared with non-POAG groups, unpaired Student's t-test. Each symbol in graphs represents datum from each cellular culture supernatant sample.

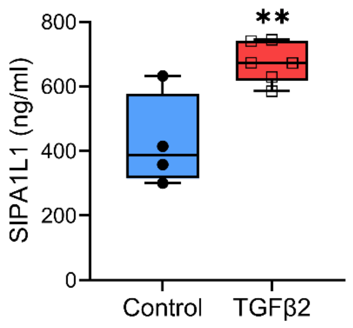

Figure S2. The cellular culture supernatant exhibits elevated SIPA1L1 concentration upon TGFβ2 induction. Cellular culture supernatant concentration of SIPA1L1 in control (n = 4) and TGFβ2 groups (n = 6) were assayed by ELISA. Data are expressed as mean ± SEM. **: P < 0.01 compared with control groups, unpaired Student's t-test. Each symbol in graphs represents datum from each cellular culture supernatant sample.

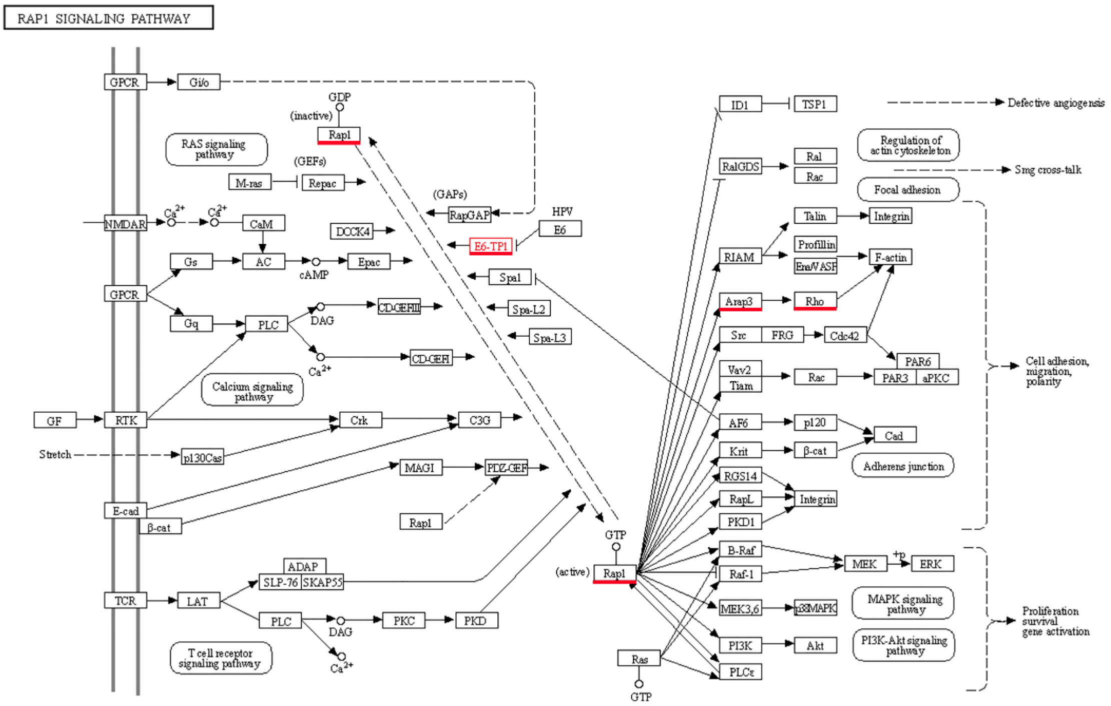

Figure S3. KEGG pathway database about SIPA1L1-Rap1-Arap3-RhoA signaling pathway. In the KEGG pathway database, SIPA1L1, alternatively known as E6-TP1, modulates the conversion of Rap1GTP (activated Rap1) to Rap1GDP (inactive Rap1). Conversely, Rap1GTP amplifies Arap3 activity, endowing Arap3 with the capacity to inhibit RhoA activation. Consequently, SIPA1L1 augments RhoA activity through the SIPA1L1-Rap1-Arap3-RhoA axis.

Table S1. Clinical details of patients

Patient ID	Age, year	Gender	Disease	Eye Laterality
1	69	Male	AACG	Left
2	59	Male	AACG	Left
3	67	Male	AACG	Left
4	53	Female	AACG	Right
5	65	Female	AACG	Left
6	53	Female	AACG	Left
7	57	Female	AACG	Left
8	53	Male	CACG	Right
9	69	Male	CACG	Right
10	63	Female	CACG	Right
11	61	Female	CACG	Right
12	65	Male	POAG	Right
13	66	Female	POAG	Right
14	70	Female	POAG	Left

CACG, Chronic angle-closure glaucoma; AACG, Acute angle-closure glaucoma; POAG, Primary open-angle glaucoma

Table S2. Sequences of SIPA1L1 siRNA

siRNA ID	siRNA Sequences Sense (5'-3')	siRNA Sequences Anti-sense (5'-3')
siSIPA1L1-1	GGACAUGUCCAAAGUGCUUTT	AAGCACUUUGGACAUGUCCTT
siSIPA1L1-2	CCGUUCAUCUCUCUGGCUUTT	AAGCCAGAGAGAUGAACGGTT
siSIPA1L1-3	CCUGGUAGAUGCUGCCAAATT	UUUGGCAGCAUCUACCAGGTT

Table S3. Primer Sequences for RT-qPCR

Target Genes (human)	Primer Sequences Forward (5'-3')	Primer Sequences Reverse (5'-3')
**SIPA1L1**	AGCCATGGCACAAGAATATAAGAA	TGCTTCACTTTAACAGAGGGCT
**FN**	TGACAAGCAGACCAGCTCAG	TTGGTGGGCTGACATTCTCC
**LN**	CTAATCCTCGGGGTTGCACA	CGCCACCCATCCTCATCAAT
**COL-IV**	GGCTGGTGAGCCAGGTTTTA	TTTGCGCCCAGGTATCCTTT
**GAPDH**	CGAGATCCCTCCAAAATCAA	GTCTTCTGGGTGGCAGTGAT
