# Supplementary material for: Effects of SIPA1L1 on trabecular meshwork extracellular matrix protein accumulation and cellular phagocytosis in POAG
Source: JCI Insight. 2024 Nov 22;9(22):e174836. doi: 10.1172/jci.insight.174836 (PMC11601898; doi:10.1172/jci.insight.174836)

Unedited blot and gel images

All unedited images of Western Blot were deposited as follows. The abbreviation of each lane was used to represent each group, in which the “C” means “Control”, “T” means “TGFβ2”, “T+si” means “TGFβ2+si-SIPA1L1”, “si” means “si-SIPA1L1” and “OE” means “Overexpressing SIPA1L1”.

Full unedited blot/gel for Figure 2

SIPA1L1(Patient1)

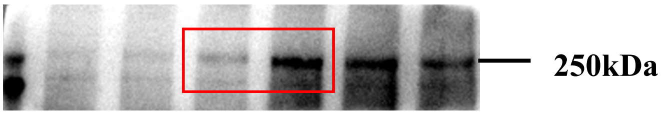

GAPDH

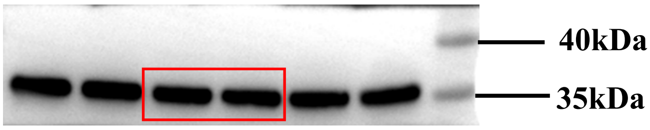

Non-POAG POAG

SIPA1L1(Patient2)

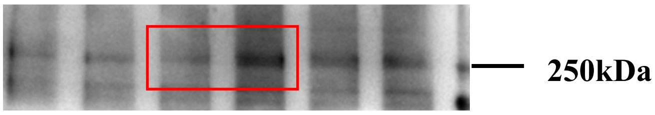

GAPDH

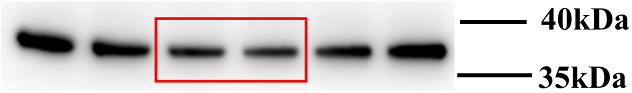

Non-POAG POAG

SIPA1L1(Patient3)

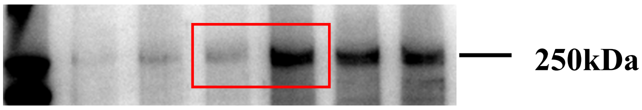

GAPDH

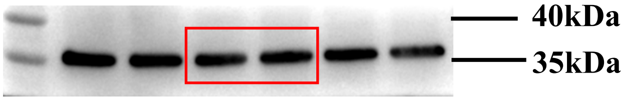

Non-POAG POAG

Full unedited blot/gel for Figure 3

SIPA1L1

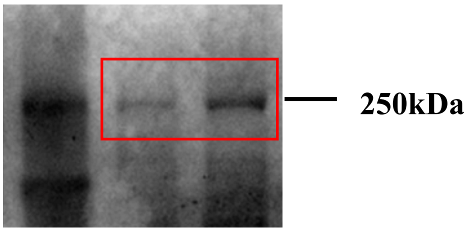

GAPDH

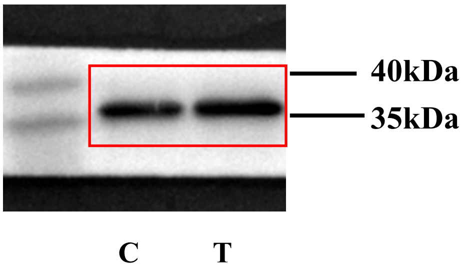

Full unedited blot/gel for Figure 4

SIPA1L1

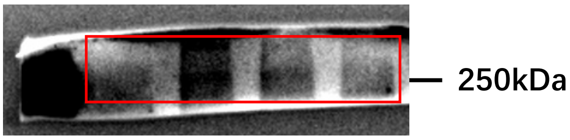

GAPDH

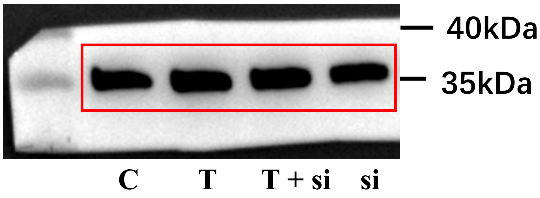

LN

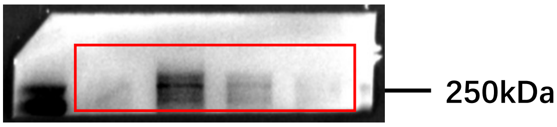

GAPDH

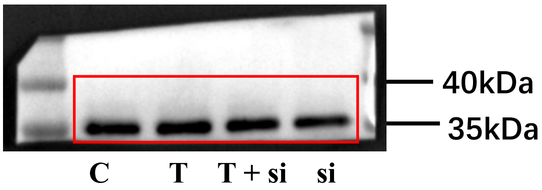

FN

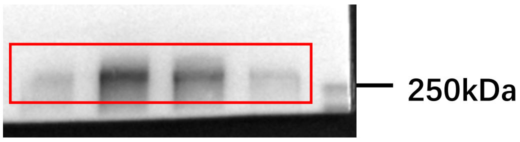

GAPDH

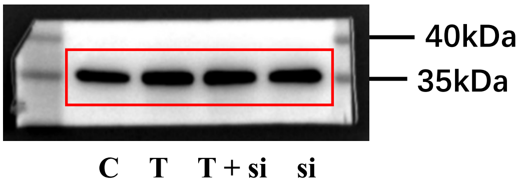

COL-IV

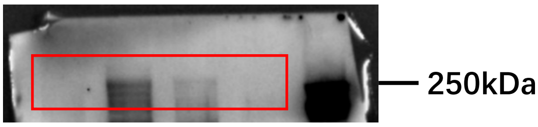

GAPDH

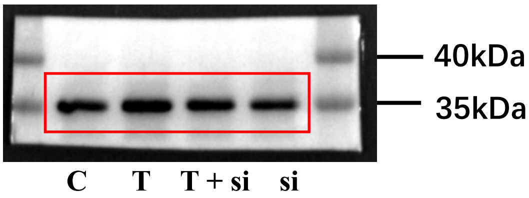

Full unedited blot/gel for Figure 5

RhoA

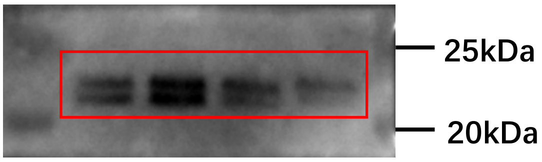

GAPDH

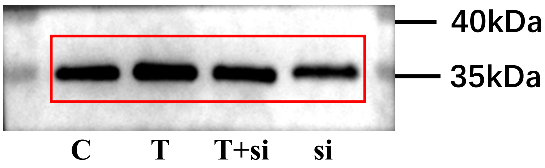

p-CFL1

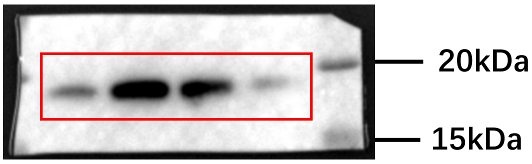

CFL1

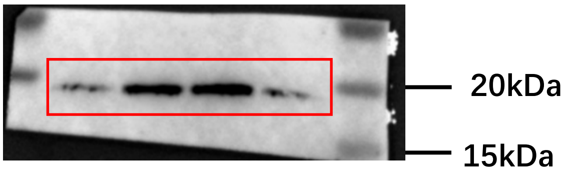

GAPDH

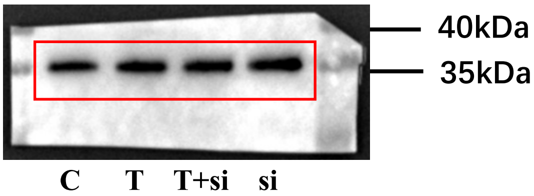

GAPDH

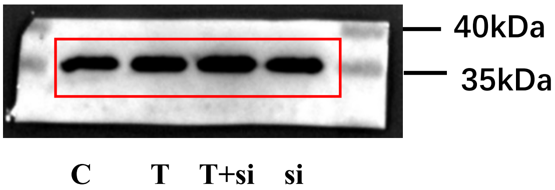

Full unedited blot/gel for Figure 6

SIPA1L1

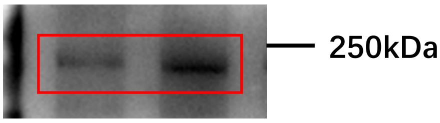

LN

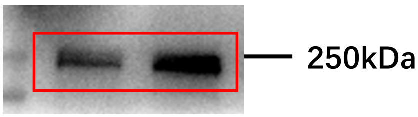

GAPDH

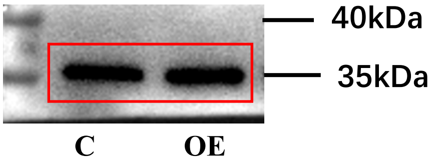

GAPDH

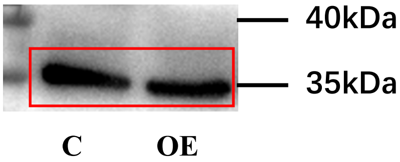

FN

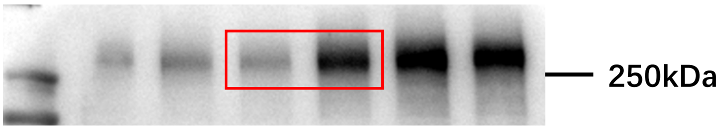

GAPDH

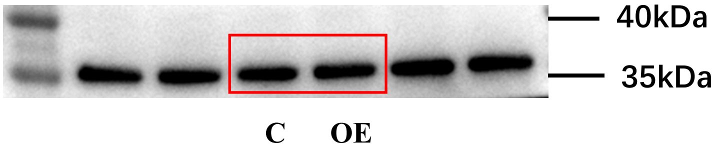

Supplement: Unedited blot and gel images [file jciinsight-9-174836-s054.pdf]
